# A Multi‐Objective Molecular Generation Method Based on Pareto Algorithm and Monte Carlo Tree Search

**DOI:** 10.1002/advs.202410640

**Published:** 2025-04-04

**Authors:** Yifei Liu, Yiheng Zhu, Jike Wang, Renling Hu, Chao Shen, Wanglin Qu, Gaoang Wang, Qun Su, Yuchen Zhu, Yu Kang, Peichen Pan, Chang‐Yu Hsieh, Tingjun Hou

**Affiliations:** ^1^ College of Pharmaceutical Sciences Zhejiang University Hangzhou Zhejiang 310058 P. R. China; ^2^ College of Computer Science and Technology Zhejiang University Hangzhou Zhejiang 310058 P. R. China

**Keywords:** drug design, molecular generation, multi‐objective optimization, pareto optimality

## Abstract

Drug discovery faces increasing challenges in identifying novel drug candidates satisfying multiple stringent objectives, such as binding affinity, protein target selectivity, and drug‐likeness. Existing optimization methods struggle with the complexity of handling numerous objectives, limiting advancements in molecular design as most algorithms are only effective for up to four optimization objectives. To overcome these limitations, the study introduces the Pareto Monte Carlo Tree Search Molecular Generation (PMMG) method, leveraging Monte Carlo Tree Search (MCTS) to efficiently uncover the Pareto Front for molecular design tasks in high‐dimensional objective space. By utilizing simplified molecular input line entry system (SMILES) to represent molecules, PMMG efficiently navigates the vast chemical space to discover molecules that exhibit multiple desirable attributes simultaneously. Numerical experiments demonstrate PMMG's superior performance, achieving a remarkable success rate of 51.65% in simultaneously optimizing seven objectives, outperforming current state‐of‐the‐art algorithms by 2.5 times. An illustrative study targeting Epidermal Growth Factor Receptor (EGFR) and Human Epidermal Growth Factor Receptor 2 (HER2) highlights PMMG's ability to generate molecules with high docking scores for target proteins and favorable predicted drug‐like properties. The results suggest that PMMG has the potential to significantly accelerate real‐world drug discovery projects involving numerous optimization objectives.

## Graphical abstract

Pareto Monte Carlo Tree Search Molecular Generation (PMMG), a molecular generation approach leveraging Monte Carlo Tree Search (MCTS) and Pareto algorithm, efficiently explores the Pareto front in high‐dimensional objective spaces for multi‐objective drug design. By representing molecules with SMILES strings, PMMG navigates the vast chemical space effectively to discover molecules exhibiting multiple desired properties simultaneously, significantly enhancing drug discovery efficiency.

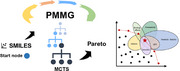

## 1 Introduction

Traditional drug design methods, encompassing target identification, lead compound discovery, and subsequent optimization, are often hampered by high costs and lengthy timelines.^[^ ^1^ ^]^ Particularly, in the initial lead discovery phase, significant investments in human and material resources are often necessary to synthesize and screen molecules. However, the advent of deep learning technology has opened exciting possibilities in the realm of drug development.^[^ ^2^ ^]^ Deep learning‐based molecular generation methods can efficiently discover a vast number of novel molecules within a short period, deep generative models like JT‐VAE^[^ ^3^ ^]^ have demonstrated remarkable capabilities in generating novel molecules by effectively modeling data distributions. Despite their potential, most models tend to generate molecules that closely resemble the training data and lack the ability to specifically target desired molecular objectives. Therefore, novel methodologies are urgently needed to develop generative models toward precise and targeted molecular optimization.

Molecular generation based on multi‐objective optimization has emerged as a critical yet intricate element of drug discovery.^[^ ^4^ ^]^ An ideal drug candidate typically needs to satisfy a balance of multiple drug‐forming properties, including biological activity, synthetic feasibility, and favorable safety profiles. The computational challenge of simultaneously optimizing conflicting properties rapidly escalates with the number of factors. Consequently, the majority of existing studies do not comprehensively evaluate their effectiveness against all the relevant properties.^[^ ^5^ ^]^ Instead, methods such as MARS^[^ ^5g^ ^]^ have been developed and tested for optimizing only four objectives (e.g., GSK3, JNK3, QED, and SA), while REINVENT^[^ ^5f^ ^]^ has been limited to just two properties. Moreover, most multi‐objective optimization strategies often resort to simplifying the problem into the single‐dimensional problem by employing weighted summation or product transformations.^[^ ^6^ ^]^ This involves assigning weights to each objective based on its relative importance. For example, Quentin et al. used a weighted summation of molecule affinities across multiple targets to identify selective and multitarget inhibitors.^[^ ^7^ ^]^ Additionally, nonlinear weighting approaches exist, like the one implemented by Chen et al., which utilizes reward values derived from Boolean operations to create a scalarized reward function.^[^ ^8^ ^]^ However, these methodologies fall short of achieving true multi‐objective optimization. Prioritizing one property excessively can mask deficiencies in others, failing to achieve a balanced and optimal outcome. Furthermore, these approaches may restrict the exploration of chemical space, leading to the development of molecules that fail to satisfy all necessary requirements.

The concept of Pareto Optimality has been pivotal in multi‐objective optimization problems.^[^ ^9^ ^]^ It clarifies the notion of balanced optimal solutions among multiple conflicting objectives and serves as a crucial guiding principle in systematically identifying these solutions. For example, Kedar et al. successfully integrated Pareto‐based evolutionary algorithms with Bayesian optimization to optimize the synthesis of silver nanoparticles under multiple constraints, achieving high‐quality synthesis with excellent yield and minimal seed particle usage.^[^ ^10^ ^]^ Similarly, Jenna et al. demonstrated the effectiveness of Pareto algorithms in multi‐objective virtual screening, significantly increasing the efficiency of discovering optimal molecules by utilizing Pareto‐based acquisition functions over scalarization approaches.^[^ ^11^ ^]^ Furthermore, Pareto Optimality has been successfully applied in molecular design. Julia et al.^[^ ^12^ ^]^ combined a generative deep learning model with a supervised deep learning model to navigate the search space and discover molecules with Pareto‐Optimal properties.

In this study, we develop the PMMG (Pareto Monte Carlo tree search Molecular Generation) algorithm for molecular generation, which utilizes a trained recurrent neural network (RNN) model as a molecular generator guided by Monte Carlo tree search (MCTS).^[^ ^13^ ^]^ MCTS continuously refines and optimizes the search direction based on the Pareto principle, exploring the vast chemical space to identify the Pareto Frontier. This approach achieves molecular generation under multiple objective constraints, yielding more satisfactory results compared to some state‐of‐the‐art optimization methods. To further validate the effectiveness of PMMG in multi‐objective optimization, we conducted a novel dual‐target ligand design targeting EGFR and HER2, which are crucial targets for treating cancers such as lung cancer and breast cancer.^[^ ^14^ ^]^ During the design process, we prioritize not only the biological inhibitory activity of molecules but also ADMET properties, drug‐likeness, and synthesizability. As a result, we successfully identified several promising compounds with predictive properties comparable to or even surpassing those of lapatinib, which holds significant implications for multi‐objective drug design.

## 2 Results and Discussion

### 2.1 Architecture of PMMG

**Figure 1:**
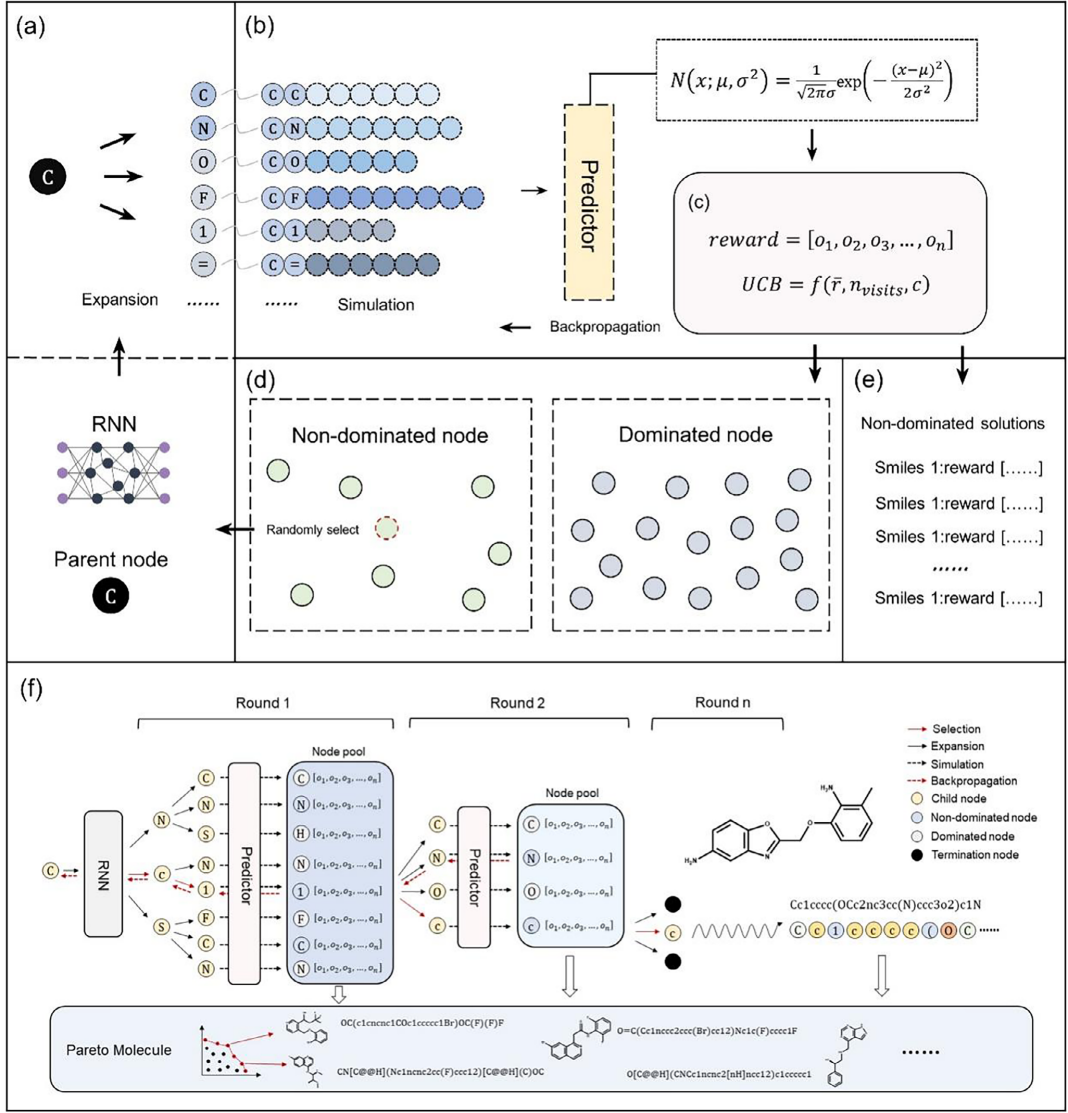
Workflow of PMMG. a) Expansion: Expand the parent node to obtain its corresponding child nodes. b) Simulation: Simulate all child nodes, predict properties, and identify all non‐dominated nodes among them. c) Backpropagation: Calculates the Upper Confidence Bound (UCB) for each node based on simulation results and updates node states through backpropagation. d) Selection: Randomly select a non‐dominated child node from the pool of non‐dominated nodes as the parent node, where a non‐dominated node is defined as one that is not inferior to any other node in at least one property. e) Non‐dominated solutions: Collect sampled molecules throughout the process, eliminate dominated molecules, and obtain the non‐dominated solution set. f) The overall workflow of PMMG.

PMMG consists of two parts: a molecular generator based on RNN and a Monte Carlo search tree used to identify the Pareto Frontier. **Figure** **1** shows the detailed flowchart of PMMG. The Pareto‐based Monte Carlo Search Tree is employed to guide the RNN in generating molecules that satisfy the desired properties. Specifically, an RNN model is trained to learn the SMILES representation rules of molecules, generating molecules by predicting the probability distribution of the next token of SMILES in both the expansion and simulation steps. During the generation process, MCTS creates a search tree and iteratively performs four steps: selection, expansion, simulation, and backpropagation. Based on the Upper Confidence Bound (UCB) scores of intermediate nodes, MCTS continuously searches and selects nodes until encountering a termination symbol.

To assess the effectiveness of our proposed model PMMG, we conducted extensive experimental validation on a benchmark dataset. In our molecular generation process, we considered a total of seven objectives, including both biological and non‐biological properties. Biological activity refers to the inhibitory activity of molecules against EGFR, where a higher value indicates stronger inhibition. Additionally, we included properties such as solubility, permeability, metabolic stability, toxicity, synthetic accessibility score (SAScore),^[^ ^15^ ^]^ and quantitative estimate of drug‐likeness (QED) score,^[^ ^16^ ^]^ which are crucial properties used in drug design. Certain properties, such as protein kinase inhibition activity and solubility, need to be maximized, while others, like toxicity and SAScore, need to be minimized. Moreover, these properties have different scales, with QED ranging from [0,1] and permeability from [0100]. Following Nathan et al,^[^ ^17^ ^]^ we utilized a Gaussian modifier to normalize all objectives, transforming them to maximize their values. This setup ensures a comprehensive consideration of various properties' impacts on the performance of the molecular generation model and provides an effective evaluation of its performance in multi‐objective optimization problems.

We selected several state‐of‐the‐art methods for comparison with PMMG, categorizing them into two main groups. The first group consists of methods based on SMILES representation, including SMILES‐GA,^[^ ^17^ ^]^ a molecular generation method based on genetic algorithms that generates new molecules by mutating and crossing SMILES representation symbols; SMILES‐LSTM,^[^ ^17^ ^]^ a method that predicts the next character in partial SMILES strings using Long Short‐Term Memory (LSTM) neural networks; SMILES‐VAE,^[^ ^18^ ^]^ which employs a Variational Autoencoder (VAE) to produce molecules represented in the form of SMILES strings; and REINVENT,^[^ ^5f^ ^]^ a reinforcement learning‐based molecular generation method that tunes RNNs to generate molecules represented in SMILES. To further demonstrate the advantages of PMMG, we also selected several graph‐based molecular generation models for comparison, including MARS,^[^ ^5g^ ^]^ a multi‐objective molecular generation method based on Graph Neural Networks (GNN) and Markov chain Monte Carlo sampling (MCMC), utilizing molecular fragments to generate molecules; Graph‐MCTS,^[^ ^19^ ^]^ a graph‐based Monte Carlo Tree Search algorithm that generates molecules in a 2D manner atom by atom; and Graph‐GA,^[^ ^19^ ^]^ a graph‐based genetic algorithm that optimizes and generates new molecules at a 2D level by crossing molecular fragments and mutating atoms or fragments.

We generated 10 000 molecules under the same conditions using each method and compared the proposed method with the baselines based on the following evaluation metrics: Hypervolume Indicator (HV), Success Rate (SR), and Diversity (Div). Except for PMMG, the results of other baselines were obtained by running the publicly available code provided by Gao et al.^[^ ^20^ ^]^ and Nathan et al.^[^ ^17^ ^]^ The predictors for bioactivity (specifically for EGFR and HER2), membrane permeability, metabolic stability, solubility, and acute toxicity were developed by Yoshizawa et al.^[^ ^6^, ^21^ ^]^ The objectives optimized were normalized to the range [0,1].

### 2.2 Performance of PMMG

The results are summarized in **Table** **1**. Overall, PMMG outperformed all baselines in three key metrics: HV, SR, and Div. Regarding the HV metric, the Pareto front generated by our algorithm achieved an HV of 0.569, outperforming the best‐performing baseline by 31.4%. This indicates that PMMG more thoroughly explored the chemical space's Pareto frontiers, demonstrating the greater potential for generating molecules that excel across multiple objectives. In terms of the SR metric, the success rate of molecules generated by PMMG reached an impressive 51.65%, far exceeding the results of the other baselines by 2.5 times.

**Table 1:** Comparison of eight different molecule generation methods under the scenario of optimizing seven targets simultaneously.

Method	HV	SR	Div
PMMG	0.569 ± 0.054	51.65% ± 0.78%	0.930 ± 0.005
SMILES_GA	0.184 ± 0.021	3.02% ± 0.12%	0.889 ± 0.003
SMILES_LSTM	0.433 ± 0.027	14.66% ± 0.45%	0.915 ± 0.009
SMILES_VAE	0.134 ± 0.061	8.58% ± 1.36%	0.906 ± 0.011
REINVENT	0.329 ± 0.047	9.63% ± 0.29%	0.915 ± 0.018
MARS	0.275 ± 0.066	12.72% ± 0.41%	0.913 ± 0.006
Graph_GA	0.199 ± 0.009	6.77% ± 0.33%	0.896 ± 0.008
Graph_MCTS	0.237 ± 0.039	2.38% ± 0.11%	0.924 ± 0.001

These findings suggest that in the simultaneous optimization of seven objectives, other methods struggled to produce a significant number of performant molecules meeting all property criteria, whereas PMMG successfully discovered more such molecules, approximately one for every two molecules generated. Regarding molecular diversity, although the diversity of molecules generated by several methods was comparable, our method still held a slight advantage. The majority of the generated molecules showed Tanimoto similarity coefficients below 0.7, indicating high structural novelty. This phenomenon may be attributed to the tendency of other methods to become confined within a specific property dimension while attempting to optimize multiple objectives simultaneously, leading to the generation of numerous structurally similar molecules. In contrast, our method effectively distributes molecules across multidimensional Pareto frontiers, with each molecule exhibiting advantages in different dimensions. This allows for the generation of a diverse array of molecular structures.

### 2.3 Performance on Individual Objectives

**Figure 2:**
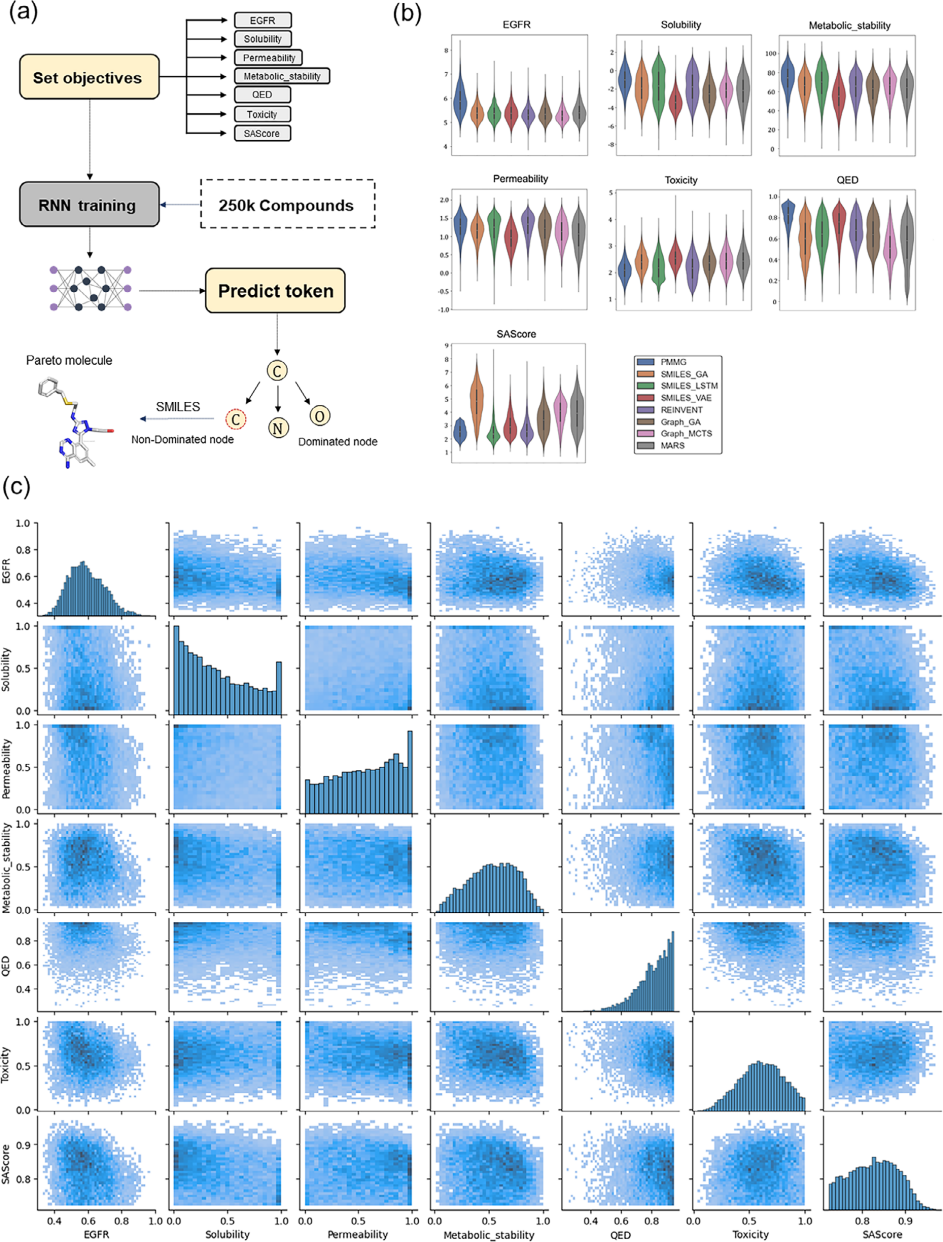
Performance on multi‐objective optimization. a) The framework of PMMG in experiments. b) The distribution of the molecules generated by each method on seven objectives. Each figure represents the distribution for a different target: EGFR, Solubility, Metabolic Stability, Permeability, Toxicity, QED, and SAScore. In each figure, the different colors of the violin plots represent seven different methods, including PMMG and six baselines. The vertical axis indicates the score range for each target. The width and length of the violin plots represent the enrichment of molecules within the corresponding score range. c) Pairwise scatter plots for each objective pair.

Next, we analyze the performance from the perspective of individual targets. The distribution of all molecules generated by each method across each property target is depicted in **Figure** **2**. Notably, PMMG demonstrates significant advantages over other baselines, particularly in terms of inhibitory activity against EGFR. For properties such as solubility, permeability, and metabolic stability, PMMG yields results comparable to the best‐performing baseline method. In terms of toxicity and synthesizability, lower scores indicate better properties. Over 90% of the molecules generated by PMMG fall below the toxicity threshold of 3.0 and have synthesizability scores below 4.0. This demonstrates a significant advantage over methods such as SMILES_GA and MARS. Upon comprehensive examination of the molecular distribution, it is evident that PMMG generates molecules predominantly within higher score ranges. This indicates that our method achieves results across all seven targets that are comparable to or surpass those of the baseline methods.

**Figure 3:**
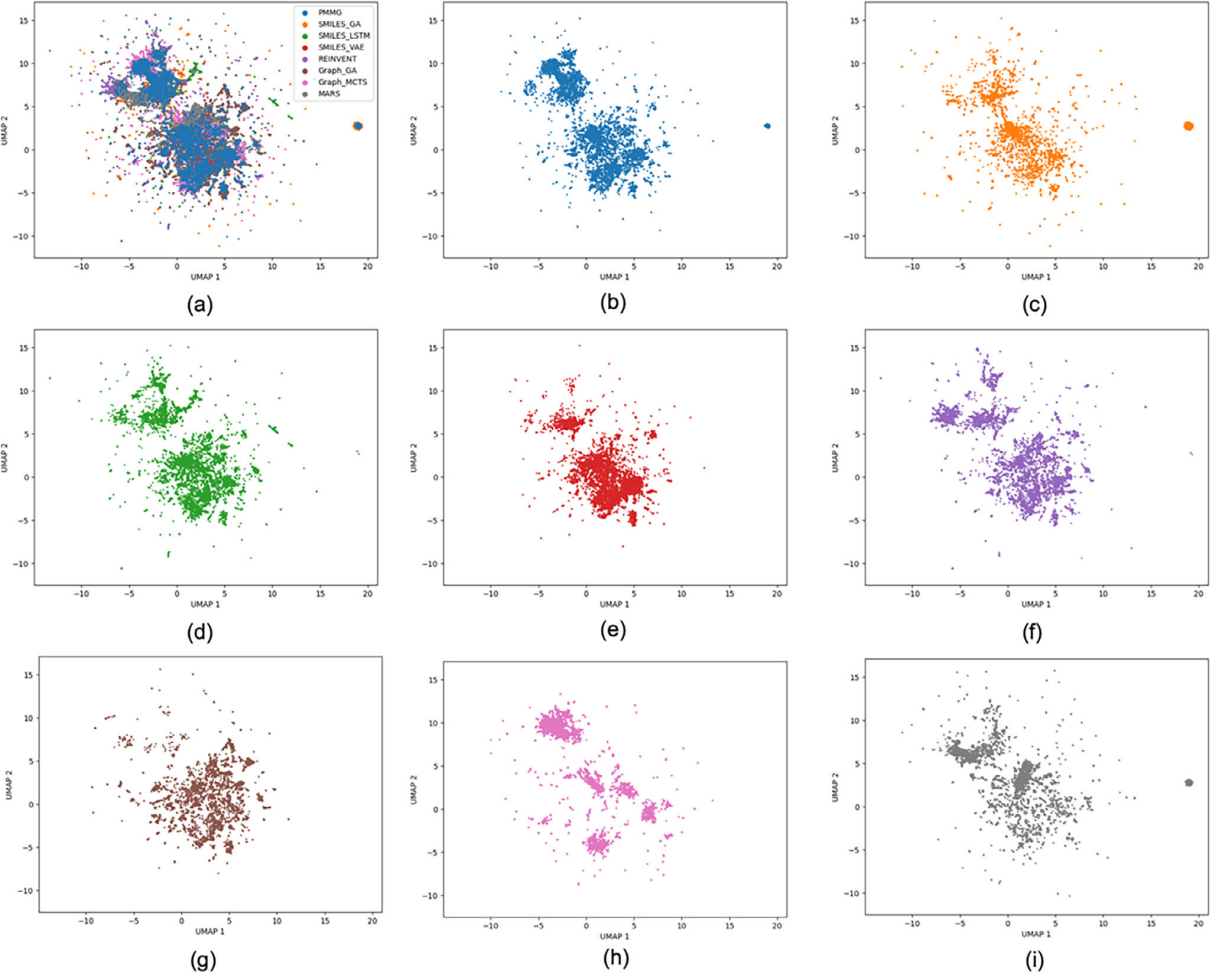
UMAP plot of molecules. a) The comparison of molecular distributions generated by the eight methods. b–i) The UMAP distribution maps of the molecules generated by PMMG, SMILES_GA, SMILES_LSTM, SMILES_VAE, REINVENT, Graph_GA, Graph_MCTS, and MARS, respectively.

The generated molecules exhibit a molecular weight range of 300 to 500 Daltons, with an average molecular weight of 400 Daltons. This distribution is relatively uniform, with no significant clustering or deviation. The molecules encompass a variety of structural scaffolds, including aromatic rings, heterocycles, and fused rings, indicating effective exploration of diverse chemical spaces during the generation process. Furthermore, these molecules feature an array of functional groups, such as hydroxyl, amino, and carboxyl groups, which are crucial for enhancing binding affinity and pharmacokinetic properties. To visualize the diversity of molecular structures, we employed Uniform Manifold Approximation and Projection (UMAP)^[^ ^22^ ^]^ for our analysis. UMAP maps molecular structures onto a 2D plane based on their molecular fingerprints. As shown in **Figure** **3**, the molecular distributions generated by the various models exhibit significant similarity. Notably, the molecules produced by the PMMG model are able to cover the vast majority of the chemical space explored by other models, which aligns well with the diversity (div) results presented in Table 1, further confirming that the outcomes are not attributable to random chance. Overall, PMMG exhibits the capability to maintain molecular structural diversity while achieving excellent optimization performance.

In addition, the synthetic accessibility of molecules is a crucial factor when evaluating the practical applicability of generative models. In addition to assessing synthetic‐related properties such as QED and SAScore for the target molecules, we performed a deeper analysis using the retro‐synthetic tool AiZynthFinder,^[^ ^23^ ^]^ which evaluates the synthesizability of each molecule and proposes feasible synthetic routes. For this evaluation, we randomly selected 100 molecules generated by each model and calculated the percentage of synthesizable molecules. This evaluation was repeated three times to ensure robustness. As shown in **Table** **2**, the average synthetic accessibility of molecules generated by PMMG reached 74.33%, second only to REINVENT, placing PMMG among the leading state‐of‐the‐art models. This result further highlights the practical utility of PMMG in real‐world scenarios.

**Table 2:** Analysis of the synthesizability of model‐generated molecules using AiZynthFinder.

Method	Synthesis Success Rate
PMMG	74.33 ± 2.03
SMILES_GA	45.67 ± 2.31
SMILES_LSTM	71.78 ± 1.09
SMILES_VAE	55.80 ± 1.33
REINVENT	76.34 ± 0.46
MARS	41.05 ± 1.53
Graph_GA	43.85 ± 1.16
Graph_MCTS	31.33 ± 0.87

Overall, PMMG's ability to generate molecules with a wide range of molecular weights and diverse structural features, while maintaining consistent quality under varying conditions, underscores its potential as a powerful tool for drug discovery. This capability to explore broad chemical space and produce stable, high‐affinity molecules positions it as a valuable asset in the development of new therapeutic agents.

### 2.4 Property Analysis of Top‐Ranked Molecules

**Figure 4:**
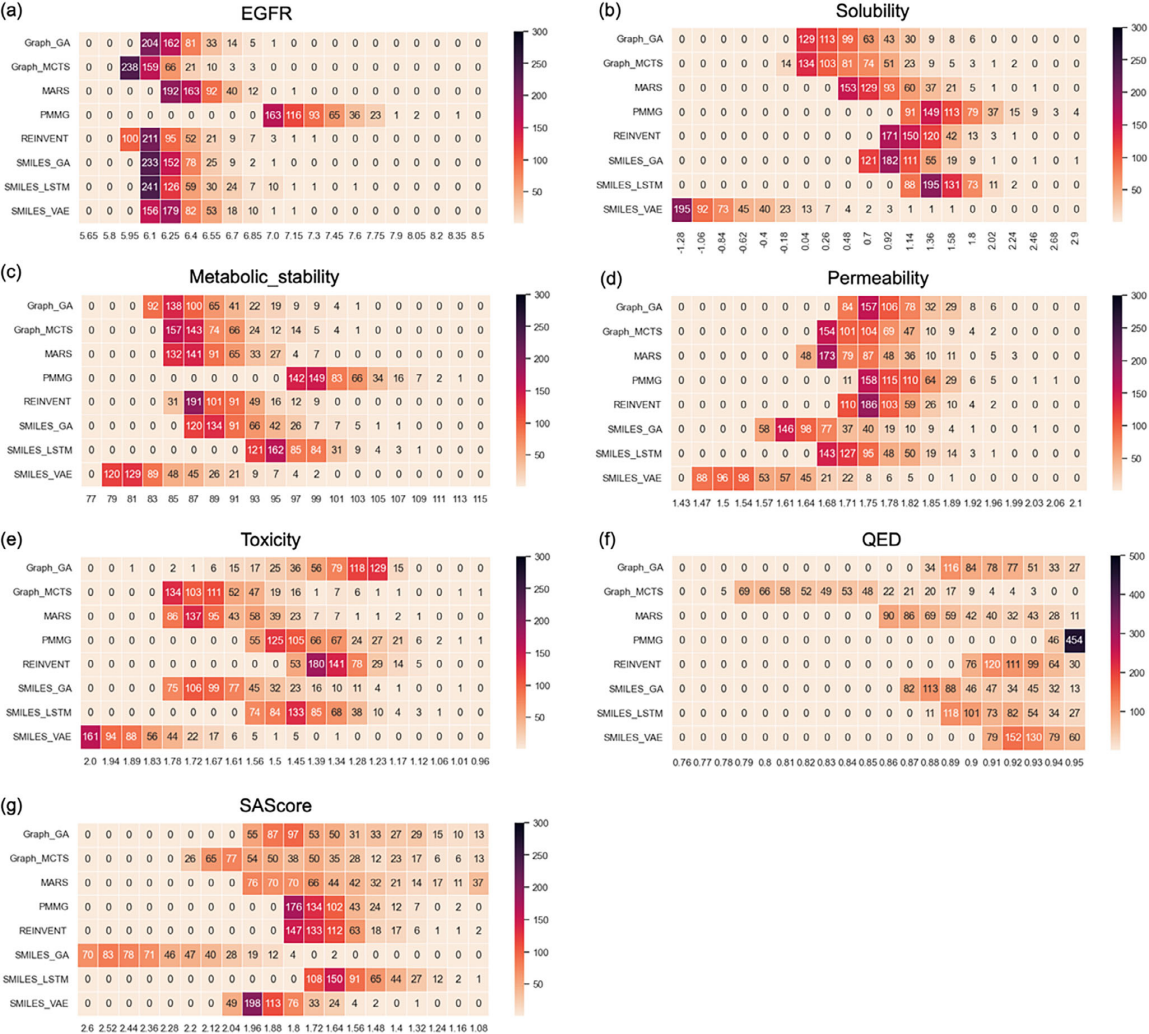
The distribution of the top‐ranked 500 molecules generated by each method on seven objectives. Each figure represents the property distribution of the top 500 molecules for each target: a) EGFR, b) Solubility, c) Metabolic Stability, d) Permeability, e) Toxicity, f) QED, and g) SAScore. In each figure, the vertical axis represents different methods, including PMMG and six baselines, and the horizontal axis represents the property distribution scores for the target. In the heatmap, the number in each cell indicates the number of molecules in that range, with darker colors representing higher numbers of molecules.

Furthermore, we analyzed the distribution of the top 500 ranked molecules produced by each method for each target, as shown in **Figure** **4**. Apart from SAScore, where methods like Graph_GA, Graph_MCTS, and MARS exhibit a more favorable distribution, PMMG demonstrates absolute superiority across most objectives, especially in terms of inhibitory activity against EGFR, metabolic stability, and QED. In summary, under the scenario of optimizing multiple targets simultaneously, PMMG consistently identifies molecules with more advantageous properties on individual targets, providing a partial explanation for the overall higher success rate of molecules from a single‐objective perspective.

Among these 500 molecules, we identified several structural scaffolds with promising pharmacological activity, such as benzothiazole, indole, and quinoline. These scaffolds are prevalent in many known drugs and are associated with favorable pharmacological and pharmacokinetic properties. In addition to these core scaffolds, the generated molecules also feature a variety of functional groups that are critical for enhancing both binding affinity and pharmacokinetic properties. Functional groups such as hydroxyl, amino, and carboxyl play significant roles in the interaction between drug molecules and their biological targets. Hydroxyl groups, for instance, can form hydrogen bonds with target proteins, improving binding specificity and strength. Amino groups are known to increase solubility and enhance the ability of molecules to interact with various biological environments, which is essential for effective drug distribution and absorption. Carboxyl groups contribute to the overall stability and reactivity of the molecules, facilitating stronger interaction with target sites and often improving the metabolic stability of drug candidates. Furthermore, the presence of these functional groups and scaffolds suggests that the generated molecules not only have high binding affinity but also possess favorable drug‐like properties. This dual advantage underscores the potential of these molecules to be developed into effective therapeutic agents. The diversity and richness of the chemical space explored by these 500 molecules provide a robust foundation for subsequent structural optimization and new drug development, aiming to enhance efficacy and reduce potential off‐target effects. By leveraging advanced computational methods and integrating predictive properties, we can streamline the identification of high‐potential candidates, ultimately accelerating the drug discovery process and improving the success rate of developing new therapeutics.

### 2.5 Design of EGFR/HER2 Dual‐Target Inhibitors

EGFR (Epidermal Growth Factor Receptor) is a receptor tyrosine kinase located on the cell membrane, involved in regulating cell growth, differentiation, and survival.^[^ ^14c^ ^]^ In various cancers, EGFR is overexpressed or mutated, leading to abnormal proliferation of cancer cells, thus making it a significant therapeutic target. HER2 (Human Epidermal Growth Factor Receptor 2), also known as ERBB2, is an important cell surface receptor frequently overexpressed in tumors such as breast cancer.^[^ ^24^ ^]^ Recent studies have shown that EGFR and HER2 can be interrelated and synergistically interact in some cancers. Therefore, simultaneous inhibition of both receptors is considered to be more effective for blocking related signaling pathways. Currently, Lapatinib^[^ ^25^ ^]^ is the only FDA‐approved dual EGFR/HER2 inhibitor drug.

In this design scenario, we consider EGFR and HER2 as two target proteins for optimization, while solubility, permeability, metabolic stability, toxicity, SAScore, and QED as other biochemical objectives. We first applied the criteria outlined in **Table** **3** to screen all the molecules, ultimately selecting 2000 molecules. The representative molecules from this selection are presented in **Table** **4**. These molecules exhibit predicted inhibitory activity against EGFR and HER2 above 6.0, along with satisfactory scores in other ADMET properties, drug‐likeness, and synthetic accessibility. Consistent with previous studies,^[^ ^26^ ^]^ the most potent EGFR/HER2 dual inhibitors typically feature a pyrimidine core structure. In the case of de novo generation, the majority of molecules in Table 4 also possess a pyrimidine core structure, demonstrating the scientific validity and effectiveness of the generation method.

**Table 3:** Criteria of objectives.

Objective	Criteria
EGFR	>5.5
HER2	>5.5
Solubility	>−3.5
Permeability	>0.4
Metabolic stability	>45
Toxicity	<3
QED	>0.6
SAScore	<4

**Table 4:** Examples of Molecules with Predicted Properties Generated by PMMG.

Molecule	EGFR	HER2	Solubility	Permeability	Metabolic_stability	Toxicity	QED	SAScore
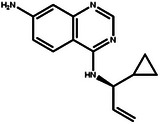	7.57	6.73	−2.17	0.91	63.70	2.45	0.64	3.26
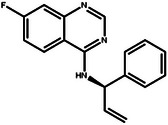	7.64	6.43	−3.45	1.19	70.02	2.94	0.73	2.81
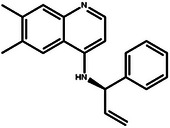	7.48	6.75	−3.42	1.48	67.38	2.82	0.71	2.85
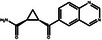	7.69	5.82	−2.29	1.36	66.47	2.21	0.81	3.24
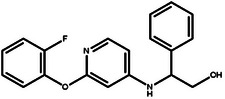	7.04	7.01	−2.55	0.87	74.66	2.57	0.71	2.56
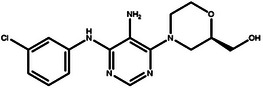	7.12	6.88	−2.59	0.42	71.20	2.99	0.78	2.86
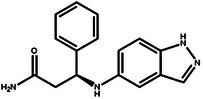	6.60	7.10	−2.62	0.45	63.46	2.18	0.67	2.62
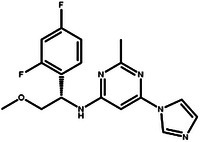	6.87	6.62	−2.09	1.23	64.55	2.44	0.74	3.17
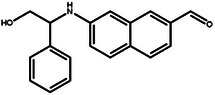	6.01	7.26	−2.50	0.80	58.30	2.30	0.70	2.49
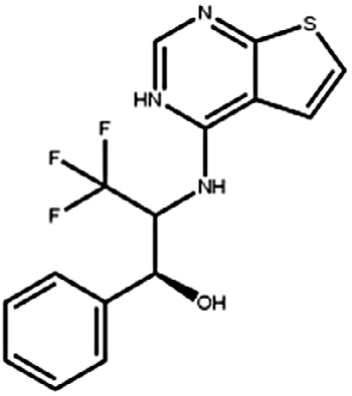	7.20	6.17	−1.90	1.10	61.18	2.85	0.76	3.36

Subsequently, we employed Glide^[^ ^27^ ^]^ to dock the 2000 molecules selected in the previous step against EGFR and HER2, followed by property prediction using ADMETlab2.0.^[^ ^28^ ^]^ We evaluated molecular binding affinity based on the ranking of Glide docking scores, where lower scores typically indicate better affinity. According to ADMETlab2.0, LogP denotes the logarithm of aqueous solubility value, while Metabolism indicates the likelihood of a molecule serving as a substrate for enzymes such as CYP 1A2/2C19/2C9/2D6/3A4, reflecting a molecule's metabolic stability. Permeability is assessed using Caco‐2 cell permeability to represent drug permeability. SAScore and QED respectively represent the synthetic accessibility and drug‐likeness of molecules.

To evaluate the binding affinity of the generated molecules to EGFR and HER2, we first conducted a statistical analysis of the docking scores for each molecule. Figure 6 illustrates that 11.28% of the molecules generated by PMMG achieved scores lower than lapatinib with a docking score of −7.88 on the EGFR target, while the docking scores of 9.72% molecules were lower than that of lapatinib on the HER2 target with a docking score of −9.30. Notably, 2.96% of the molecules outperformed lapatinib on both targets. For the EGFR target, Figure 6 shows that the majority of the molecules have docking scores concentrated between −8 and −4, with a median score of ≈−6. Some molecules with lower docking scores were prioritized for further analysis. Similarly, for the HER2 target, the docking scores of the generated molecules mainly ranged from −7 to −10. The median docking score was ≈−8, slightly higher than the median score for the EGFR target.

**Figure 5:**
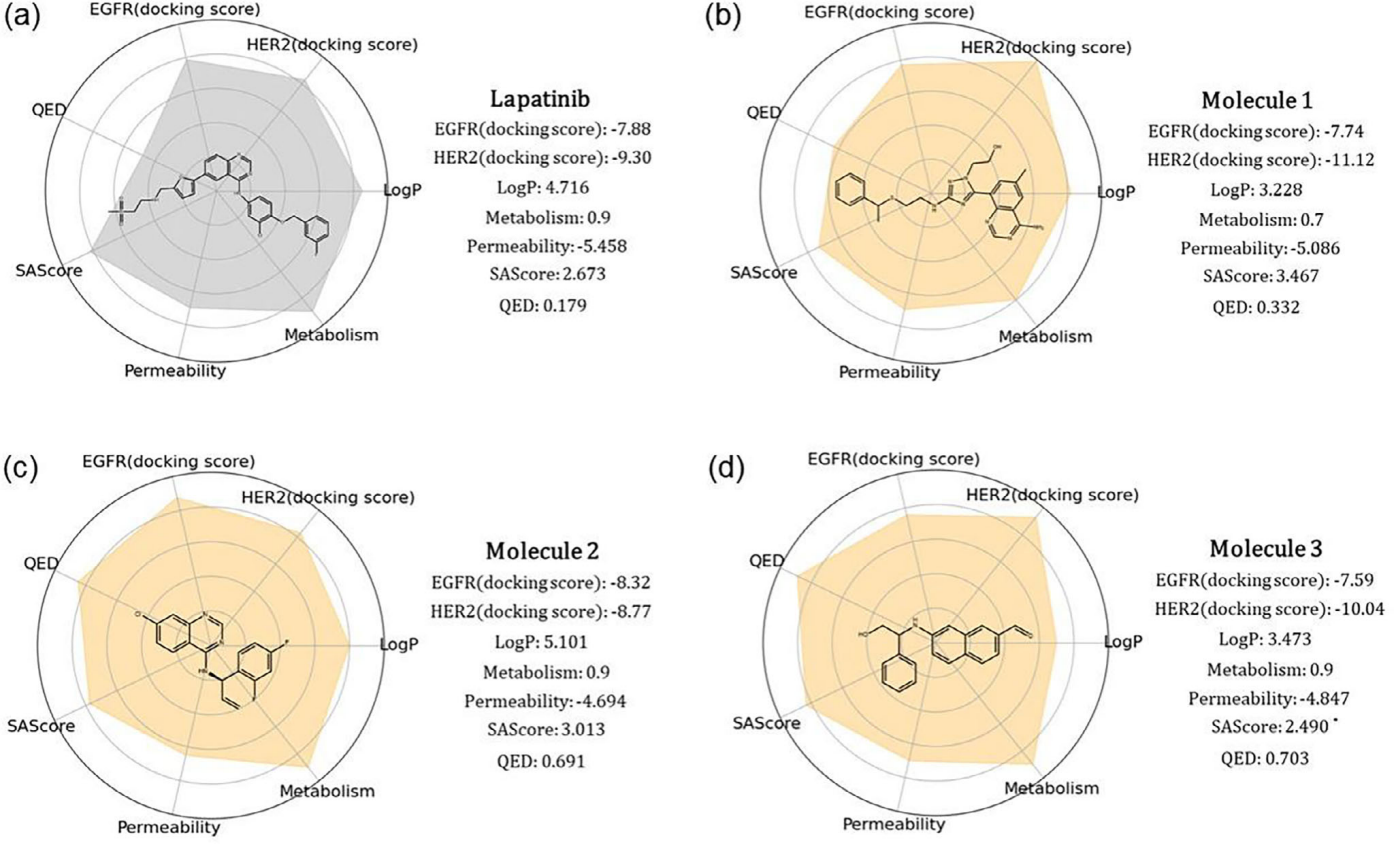
Predicted properties of Example Molecules. a) The docking score and predicted properties of lapatinib. b) The docking score and predicted properties of Molecule 1. c) The docking score and predicted properties of Molecule 2. d) The docking score and predicted properties of Molecule 3. The figures include seven properties: EGFR docking score, HER2 docking score, LogP, Metabolism, Permeability, SAScore, and QED. The radar charts display their performance for each objective.

Based on the results from screening, we further identified and analyzed the top‐ranking molecules with high docking scores for EGFR and HER2 by considering additional six predictive properties. **Figure** **5** illustrates examples of molecules based on the results, where the molecules exhibit docking scores comparable to or even surpassing lapatinib, indicating their potential effectiveness against both targets. Additionally, these molecules demonstrate similar or superior predicted properties compared to lapatinib, including LogP, membrane permeability, metabolic stability, synthetic accessibility, and drug‐likeness, suggesting their potential as promising drug candidates.

### 2.6 Docking Conformation

**Figure 6:**
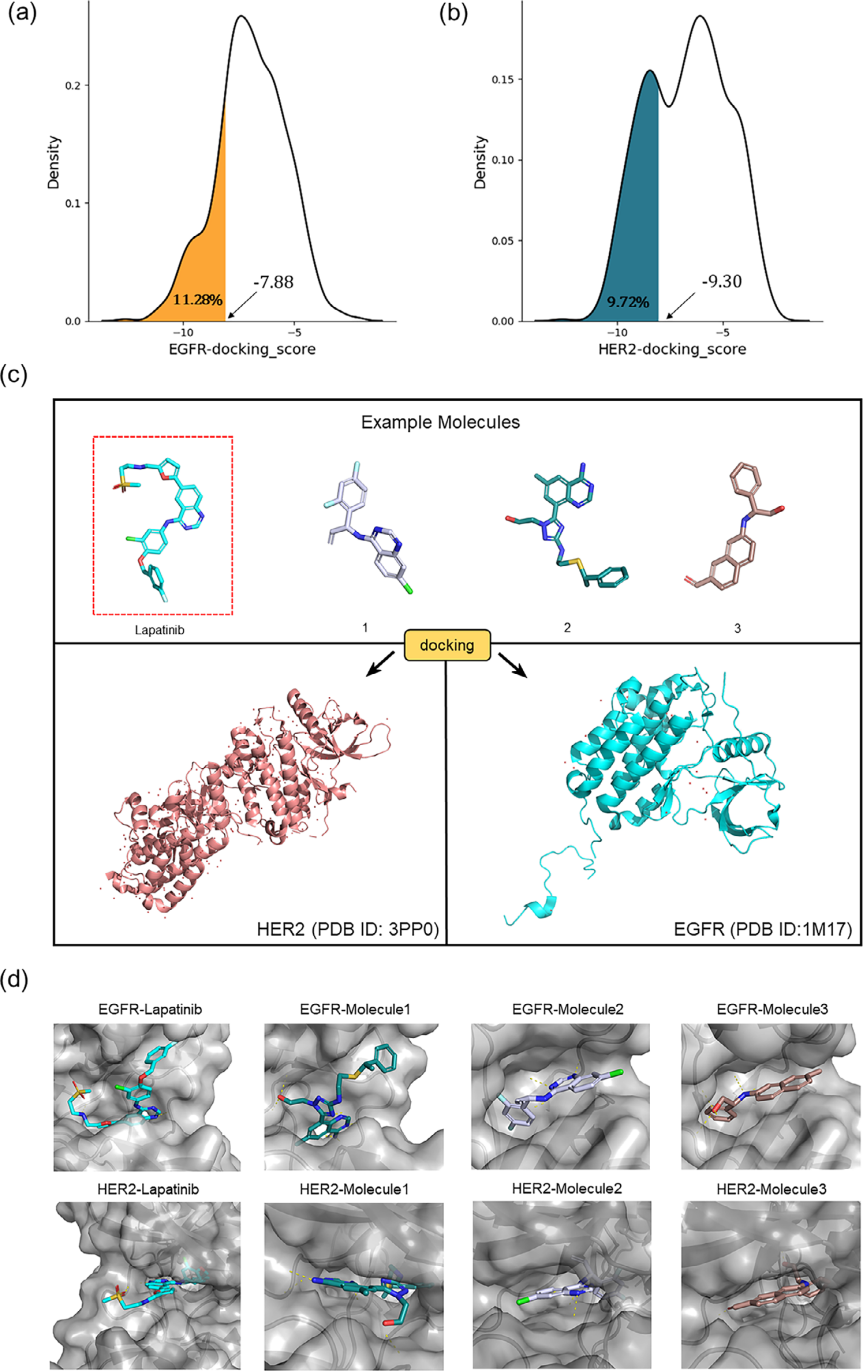
Docking results. Distribution of Docking Scores for PMMG‐Generated Molecules on a) EGFR and b) HER2. The horizontal axis in the figures represents docking scores, and the vertical axis represents density distribution. In (a), the shaded area (orange) represents the proportion of molecules with docking scores <−7.88. In (b), the shaded area (dark green) represents the proportion of molecules with docking scores <−9.30. The scores −7.88 and −9.30 are the docking scores of Lapatinib for the EGFR and HER2 targets, respectively. c) Docking of example molecules; the molecule circled in the red box is the reference molecule, lapatinib. d) Docking conformations of Molecules 1, 2, 3, and Lapatinib with EGFR and HER2. The four molecules in the row from left to right are Lapatinib, Molecule 1, Molecule 2, and Molecule 3, docked with EGFR (1M17) and HER2 (3PP0), positioned within the protein pocket.

**Figure** **6** illustrates the docking conformations of three molecules and lapatinib binding to EGFR and HER2. Structurally, Molecule 1 exhibits a conformation similar to lapatinib, with a docking score of −7.74 against EGFR. The docking poses indicate that Molecules 1, 2, and 3 can effectively bind into the active site of EGFR, stabilizing through multiple hydrogen bonds and hydrophobic interactions. Notably, the hydroxyl group of Molecule 1 forms a strong hydrogen bond with the critical amino acid ASP863 in EGFR, which may help to significantly enhance binding affinity. Molecule 1 also demonstrates a lower docking score of −11.12 against HER2, suggesting that it may exhibit a relatively high binding affinity for HER2. Docking simulations reveal that the aromatic ring structure of Molecule 1 interacts with the hydrophobic pocket of HER2, while its amino group forms a stable hydrogen bond with the key residue MET801. This flexible structure allows it to adapt to the active sites of both targets. Molecules 2 and 3 also form effective hydrophobic interactions and hydrogen bonds with both targets through their aromatic rings and polar groups, showcasing excellent multi‐target binding capabilities. By analyzing the structural characteristics of high‐affinity dual‐target molecules, we found that these molecules typically possess good molecular flexibility and multifunctional groups, enabling them to meet the binding requirements of different targets. These findings provide important guiding principles for the design of multi‐target drugs.

### 2.7 Absolute Binding Free Energy (ABFE) Calculations

To further validate the effectiveness of our method, the absolute binding free energies for Lapatinib and the three designed molecules against both targets were calculated using the Double Decoupling Method (DDM)^[^ ^29^ ^]^ implemented in NAMD3.^[^ ^30^ ^]^ All simulation inputs were automatically generated by BFEE23. The bound and unbound states were divided into 200 λ and 100 λ windows, respectively. Each window performed a 0.4 ns of equilibration and 2 ns of alchemical transformation, incorporating the DWS FEP and WTM‐eABF algorithms. The absolute binding free energies were postprocessed using the BAR estimator in the BFEE2.^[^ ^31^ ^]^ More details of the preparation, simulation, and post‐processing are provided in SI. The experimental binding affinities for lapatinib were calculated based on its *K*_i_ value.^[^ ^32^ ^]^ As shown in **Table** **5**, for the EGFR target, molecules 1 (ΔG = −8.58 ± 4.06kcal mol^−1^) and 3 (ΔG = −7.42 ± 0.52 kcal mol^−1^) exhibit binding free energies lower than that of the positive drug lapatinib. However, both molecules still have potential for further optimization and structural modifications to improve their binding affinities. In contrast, molecule 2 (ΔG = −0.30 ± 0.54 kcal mol^−1^) shows significantly weaker binding than lapatinib and is unsuitable for the EGFR target. For the HER2 target, molecules 1 (ΔG = −12.85 ± 0.58 kcal mol^−1^), 2 (ΔG = −11.23 ± 0.67 kcal mol^−1^), and 3 (ΔG = −13.86 ± 0.49 kcal mol^−1^) all outperform lapatinib (ΔG = −10.82 kcal mol^−1^), with molecule 3 exhibiting the best binding affinity and computational stability. Overall, molecules 1 and 3 are identified as promising candidate molecules, particularly with significant advantages in HER2 targeting.

**Table 5:** The predicted absolute binding free energies for lapatinib and three designed molecules.

	EGFR	HER2
Lapatinib	−11.70 kcal mol^−1^	−10.82 kcal mol^−1^
Molecule 1	−8.58 ± 4.06 kcal mol^−1^	−12.85 ± 0.58 kcal mol^−1^
Molecule 2	−0.30± 0.54 kcal mol^−1^	−11.23 ± 0.67 kcal mol^−1^
Molecule 3	−7.42 ± 0.52 kcal mol^−1^	−13.86 ± 0.49 kcal mol^−1^

### 2.8 Genscore Evaluation

In addition to molecular docking, we further assessed the affinity of molecules to their targets using Genscore,^[^ ^33^ ^]^ a machine learning‐based protein‐ligand scoring framework developed by our group. Genscore has demonstrated exceptional performance in comparing the relative binding free energies of different molecules and has consistently shown strong predictive power across several benchmark datasets.^[^ ^33^ ^]^ Higher scores correlate with better potential binding affinity. For this evaluation, we employed the top‐performing GatedGCN_1.0 model, and the results are summarized in **Table** **6**. For the EGFR target, molecules 2 and 3 achieved scores comparable to that of the reference compound, lapatinib. For the HER2 target, all three molecules significantly outperformed lapatinib, suggesting that they may form stronger binding with the targets than the reference molecule.

**Table 6:** Rescoring Results Using GenScore (GatedGCN_1.0).

	EGFR↑	HER2↑
Lapatinib	31.43	20.80
Molecule 1	18.55	37.90
Molecule 2	37.74	31.71
Molecule 3	30.34	33.90

## 3 Discussion

Most multi‐objective optimization methods exhibit diminishing performance with an increasing number of objectives.^[^ ^34^ ^]^ In the case of PMMG, this increase in objectives correlates with an expansion of the chemical space, leading to exponential growth in exploration volume. Consequently, with adequate time and computational resources, the model acquires increased autonomy in navigating the Pareto front, thereby alleviating the efficiency downturn linked to heightened objective complexity. This adaptability proves particularly advantageous in drug design contexts characterized by higher dimensionality, substantially augmenting PMMG's practical efficacy. Although PMMG is based on a 1D SMILES representation, its performance still outperforms some graph‐based methods that can capture molecular structural information more precisely. This highlights the potential of combining Pareto optimization with Monte Carlo Tree Search algorithm to provide powerful guidance in exploring molecular space. Furthermore, we are also considering the integration of this approach with graph‐based or 3D‐based methods to potentially enhance performance further, which remains a direction for future exploration. However, at present, we continue to believe that this method is particularly well‐suited 1D molecular representations.

Furthermore, we have developed a multi‐objective optimization algorithm for our method that utilizes feedback from reward models, such as QSAR models, during the optimization process. It can be stated that the algorithm itself is independent of the reward model, offering exceptional flexibility. As a result, the upper limit of our method's performance is largely determined by the reward model. This allows us to effortlessly integrate a multifidelity feedback mechanism, such as multifidelity Bayesian optimization (MF‐BO), into our framework, incorporating lower‐ and medium‐precision methods like FEP and docking, as well as high‐precision wet lab results. In Bayesian optimization, as more experimental feedback is incorporated, the performance of the reward model improves, allowing the method to achieve accuracy that approaches, or even becomes indistinguishable from, real experimental results. Of course, wet lab experiments and methods like FEP also incur higher costs, which highlights both the high potential and flexibility of our algorithm.

However, PMMG exhibits certain limitations. Despite the multiplicity of objectives for optimization, each objective may entail distinct requirements. While certain objectives necessitate adherence to specific criteria such as permeability and toxicity, others, such as protein target affinity, generally benefit from higher values. Thus, while effectively exploring specific vector directions in chemical space is crucial, extensive exploration along certain directions may prove futile. Another significant concern arises from PMMG's capacity to generate an extensive Pareto front comprising thousands of molecules, rendering the selection of desired molecules from this vast array challenging. Future optimization and improvements could prioritize directional exploration and sampling along specific directions of the Pareto front in the chemical space based on user demands.

Moreover, limitations inherent in MCTS, RNN, and SMILES grammar itself may affect the validity, chirality, and specificity of the generated molecules. As sequence length increases, RNNs may suffer from gradient explosion, failing to effectively capture long‐range dependencies. Therefore, alternatives such as transformers could be explored for molecular generation to address this issue. However, it is imperative to acknowledge that the adoption of these methodologies may introduce heightened algorithmic and search complexities. The integration of MCTS with neural networks requires meticulous attention to algorithm design and parameter tuning to strike a balance between exploration and exploitation within the search space.

Certainly, assessing a model's quality should not rely solely on experimental results, but computational efficiency and scalability are equally important factors. Due to the complex exploration steps and filtering mechanisms involved, PMMG may require more computational resources compared to other models. To be specific, our server is equipped with one NVIDIA A100‐SXM4‐80GB GPU and two 64‐core Intel(R) Xeon(R) Platinum 8358P CPUs @ 2.60 GHz. In practice, PMMG requires an average runtime of 16 h to generate 10 000 molecules, whereas other baseline models complete this task within 1 h. These results clearly show that PMMG has a higher computational cost. However, when considering the multi‐year timescale of drug discovery and development, such computational costs are relatively insignificant. Importantly, the experimental results demonstrate that PMMG effectively utilizes these computational resources to identify higher‐quality potential molecules, reflecting a resource utilization rate comparable to, if not better than, that of other models. Overall, the scalability and practicality of PMMG are substantiated by its performance.

## 4 Conclusion

In conclusion, we propose PMMG by integrating the principle of Pareto Optimality with MCTS for multi‐objective molecular generation, targeting high target selectivity and affinity, ADMET properties, QED, and SAScore, among others. To validate the model's effectiveness, we compare it with several state‐of‐the‐art methods. PMMG achieves superior results across all three essential metrics of multi‐objective molecular generation, i.e., HV, SR, and Div, in optimizing inhibitory activity against EGFR, solubility, permeability, metabolic stability, SAScore, and QED. The SR of the molecules reached 51.65%, significantly surpassing other methods. Subsequently, we conducted a dual‐target drug molecule design for EGFR and HER2, successfully generating molecules with docking scores and predicted properties comparable to or even surpassing lapatinib in certain properties, thereby validating the effectiveness of the model.

While there is substantial room for further development, our current version of the PMMG has achieved significant milestones in multi‐objective molecular generation. By effectively balancing and optimizing multiple molecular properties simultaneously, such as potency, selectivity, and ADMET profiles, PMMG demonstrates a robust capability to address the complex and often conflicting requirements inherent in drug discovery. Utilizing state‐of‐the‐art algorithms, our model has shown promising results in generating novel compounds that align with desired therapeutic profiles. This underscores the meaningful contribution of our model to the field of multi‐objective molecular generation, providing a versatile and powerful tool that enhances the efficiency and accuracy of identifying potential drug candidates. The achievements of PMMG pave the way for future advancements in drug design and discovery, where the integration of more sophisticated machine learning techniques and the expansion of chemical space exploration are anticipated. As the model continues to evolve, it holds the potential to revolutionize the approach to molecular generation, offering a significant advantage in the development of new therapeutics with optimized properties.

## 5 Experimental Section

### PMMG

PMMG integrates the Pareto algorithm in four aspects:

*Reward Redefinition*: The reward was redefined in the backpropagation step as a vector consisting of n dimensions, where each dimension represents one of the objectives we aim to optimize, rather than a single value obtained by the weighted sum.

(1) $$reward=\left[o_{1},o_{2},o_{3},\text{…},o_{n}\right]$$

*UCB Redefinition*: Similarly, the UCB was redefined in the selection step as an n‐dimensional vector, composed of the average reward value $\bar{r}$, total exploration counts *n_visits_*, and Exploration coefficient *c*, to guide node selection.

(2) $$UCB=f\left(\bar{r},n_{visits},c\right)$$

*Optimal Child Node Pool*: An optimal child node pool was defined for each parent node. During the simulation and backpropagation process, each child node is assigned an n‐dimensional UCB. Before the selection step, all child nodes are pairwise compared along each dimension of the UCB. If a child node is not inferior to another child node across all dimensions, it is termed as a non‐dominated node, while the latter is considered as a dominated node, both of which represent a token of SMILES that can potentially form molecules in the simulation step. Then we remove dominated child nodes and add all non‐dominated child nodes to the optimal child node pool, resulting in a set of non‐dominated solutions. During node selection, a random optimal child node from the pool is chosen for expansion.

*Optimal Molecule Pool*: We define an optimal molecule pool as the final output molecule pool. All generated molecules are compared to all molecules in the pool based on their n‐dimensional reward values. If a molecule is not dominated by any molecule in the pool, it is added to this pool. Conversely, if a molecule is dominated, it is discarded. Similarly, if an old molecule in the optimal molecule pool is dominated by a new molecule, the old molecule is also discarded.

### RNN

The RNN model utilized in the study comprises an 81‐dimensional embedding layer and two 256‐dimensional GRU layers, trained on 48 core Intel(R) Xeon(R) Gold 6240R CPUs @ 2.40 GHz. The training dataset contains ≈250 000 compounds from the ZINC database,^[^ ^35^ ^]^ with training parameters as follows: a learning rate of 0.01, a batch size of 256, and training over a total of 100 epochs.

### Pareto Optimality in Multi‐Objective Optimization

The Pareto Optimality, also known as Pareto front method, is commonly used in multi‐objective optimization. The core idea of this algorithm originates from the concept of Pareto optimality proposed by the French mathematician Vilfredo Pareto,^[^ ^9^ ^]^ which states that under given constraints, it is impossible to improve one objective without sacrificing at least one other objective. The Pareto algorithm aims to find such a set of solutions, forming the Pareto frontier, which includes all non‐dominated optimal solutions. First, some relevant concepts of the Pareto algorithm were introduced in multi‐objective optimization problems.

*Pareto dominance*: A decision vector *a* is said to Pareto dominate *b* if the performance of *a* on any objective function is no worse than that of *b*.

This concept emphasizes the balance and trade‐offs in multi‐objective optimization, as the superiority of a solution depends not only on the optimality of individual objectives but also on the relative performance across multiple objectives. By identifying Pareto dominance relationships, researchers can determine the set of non‐dominated solutions in the entire objective space, forming the Pareto frontier, which provides a more comprehensive selection of solutions for the final resolution of the problem.

*Pareto optimal solution*: In a multi‐objective optimization problem, let *x* represent a decision space containing possible solutions. For each solution *x*, there are *m* objective functions *f_1_* (*x*), *f_2_* (*x*)*……f_m_* (*x*), representing the performance under different objectives. The Pareto optimal solution can be formally defined as follows:

Suppose there are two solutions *x_1_* and *x_2_*, for all *j* = 1,2,…,*m*, it is define:

1) *x*_1_ is not inferior to *x*_2_ on objective *j*, denoted as *f_j_* (*x*_1_) *≥ f_j_* (*x*_2_).
2) There exists at least one objective *k* such that *x*_1_ is superior to *x*_2_ on objective *k*, denoted as *f_k_* (*x*_1_) > *f_k_* (*x*_2_).

If *x*_1_ satisfies the above conditions, then we say *x*_1_ Pareto dominates *x*_2_, denoted as *x*_1_≻*x*_2_.

*Pareto optimal solution set*: typically denoted as *P*∗, refers to the set of all Pareto optimal solutions in the decision space *X*. Formally, *P*∗ is a set of solutions that satisfies the following conditions:

1) For any *x*∈*X*, there exists no solution *y*∈*P*∗ such that *y*≻*x*.
2) There exists at least one solution *z*∈*P*∗ such that for any *x*∈*X*, *z*⪯*x*.

This implies that the solutions in *P*∗ cannot be dominated by any other solution on all objective functions, and at least one solution is superior to other solutions on at least one objective. Such a set of solutions forms the Pareto frontier, representing the set of non‐dominated solutions in multi‐objective optimization problems.

*Pareto frontier*: The Pareto frontier is an important concept in multi‐objective optimization problems, typically denoted as PF. It refers to a set of solutions in the decision space that cannot be dominated by any other solution on all objective functions. These solutions' objective function mappings form the Pareto optimal frontier or Pareto front face of the problem. For a two‐objective problem, the Pareto optimal frontier is typically a curve, whereas for multiple objectives, it is usually a hyper‐surface.

### Monte Carlo Tree Search

MCTS^[^ ^13^ ^]^ is a decision‐making algorithm based on the Monte Carlo method, widely used in areas such as gaming, planning, and generation. In the field of drug design, the MCTS algorithm is often employed in molecular generation models, such as ChemTS,^[^ ^36^ ^]^ where the nodes of the search tree represent different conformations or topological structures of molecular states, and the edges of the tree represent transitions from one molecular state to another, i.e., different conformations or topological structures. The action space needs to define reasonable chemical reactions or conformational transformations for expanding nodes during the search. Each node records the number of visits and accumulated rewards. In molecular generation, this can be interpreted as the frequency of selection of a conformation or topological structure and its corresponding evaluation reward. The specific steps of tree expansion in MCTS for drug design are as follows:

*Selection*: Starting from the root node, nodes are selected based on a certain selection strategy (e.g., UCB algorithm) until an unexpanded node is found. In molecular generation, node selection may be based on previous molecular generation experiences, tending to select conformations or structures that have not been sufficiently explored.

*Expansion*: Expansion is performed on the selected node by generating new molecular states. This can be achieved by introducing new conformations, topological structures, or introducing new molecules through chemical reactions.

*Simulation*: The newly generated molecules are simulated, for example, through evaluation using QSAR models, to obtain reward values for the simulated paths. This helps estimate the biological activity or drug properties of the molecules.

*Backpropagation*: The obtained reward values from the simulation are propagated back to all nodes traversed in the search tree, updating their visit counts and cumulative rewards. This reflects the impact of the quality of the newly generated molecules on all related molecular states in the search tree.

Throughout the Monte Carlo Tree Search process, the two most important steps are node selection and backpropagation. In the backpropagation process, the core is the reward value, which is typically composed of visit counts and cumulative rewards. The reward value serves as a “label” assigned to the current state during the backpropagation process. The more the properties align with our requirements and expectations, the higher the score assigned to the node state. This label continues to participate in guiding the node selection process, playing a crucial role in the exploration of the chemical space for molecular generation. In the node selection process, the upper confidence bounds (UCB) are often used to represent the advantage estimate of the action, thereby determining the quality of the node. The UCB value for the i‐th node is typically represented as follows:

(3) $$UCB_{i}=\bar{v_{i}}+c\sqrt{\frac{\ln N}{n_{i}}}$$

where $\bar{v_{i}}$ is the average value of the i‐th action, *c* is a constant, *N* is the total number of explorations, and *n_i_* is the exploration count of the current node. This includes reward values and exploration situations. The core idea of the UCB algorithm is to prioritize actions with high average rewards (exploiting known information) but relatively fewer attempts (exploring unknown information). By adjusting the parameter *c*, the trade‐off between exploration and exploitation can be balanced. The action with the highest UCB value is selected for further exploration or exploitation

### Evaluation Metrics

*Hypervolume Indicator (HV)*: *HV* measures the volume of the space dominated by the Pareto front of the solutions,^[^ ^37^ ^]^ bounded from below by the preference point (0, 0). A higher HV value means better quality of the Pareto front

(4) $$HV_{t}=volume\left(\overset{|PF_{t}|}{\underset{i=1}{⋃}}v_{i}\right)$$

*Success Rate (SR)*: The proportion of generated molecules that meet the expected values for all predicted properties. Specifically, the criteria are as follows. The criteria for standard formulation are based on the predicted values of molecules in the dataset and relevant drug molecules to establish the minimum pharmaceutical requirements.

*Diversity (Div)*: Diversity measures the degree of dissimilarity among the generated molecules using pairwise Tanimoto similarity over Morgan fingerprints, reflecting the variety of molecular structures.

(5) $$Div=\frac{2}{n\left(n-1\right)}\sum_{x\neq x^{\prime }\in G}1-sim\left(x,x^{\prime }\right)$$

### Data and Code Availability

The data and source code of this study is freely available at GitHub (https://github.com/Liuyifeii/PMMG) to allow replication of the results.

## Conflict of Interest

The authors declare no conflict of interest.

## Supporting information



Supporting Information

## Data Availability

The data that support the findings of this study are available from the corresponding author upon reasonable request.
