# Supplementary material for: A Multi‐Objective Molecular Generation Method Based on Pareto Algorithm and Monte Carlo Tree Search
Source: Adv Sci (Weinh). 2025 Apr 4;12(20):2410640. doi: 10.1002/advs.202410640 (PMC12120794; doi:10.1002/advs.202410640)
Supplement: Supplementary file 1 — Supporting Information [file ADVS-12-2410640-s001.docx]

**Supporting information**

**A Multi-objective Molecular Generation Method based on Pareto Algorithm and Monte Carlo Tree Search**

Yifei Liu^1,†^, Yiheng Zhu^2,†^, Jike Wang^1^, Renling Hu^1^, Chao Shen^1^, Wanglin Qu^1^, Gaoang Wang^1^, Qun Su^1^, Yuchen Zhu^1^, Yu Kang^1,*^, Peichen Pan^1,*^, Chang-Yu Hsieh^1,*^, Tingjun Hou^1,*^

**The calculation details of the Absolute Binding Free Energy (ABFE) calculations**

We first standardized the structures of ligands and proteins. Then, the force-field parameters, charges, and topologies of each ligand were generated using Antechamber and Parmchk2 in AmberTools. Tleap in Ambertools^S1^ was used to solvate the complex and ligand in a cubic box with the periodic boundary edge set to 10.0 Å, where the selected explicit water model was TIP3P and the counter ions were 0.15 mM NaCl. The force fields for the protein and the ligand were ff14SB^S2^ and GAFF2^S3^, respectively. Cpptraj in Ambertools^S1^ was used to perform hydrogen mass repartitioning (HMR), increasing the masses of hydrogen atoms in solutes to 3.024 amu. HMR reduces the frequency of hydrogen atom-based bond vibrations, which maintains SHAKE ^S4-S6^ stable at a time step of 4 fs. Finally, the input of simulations was generated using the BFEE2^S7^.

After establishing the systems, all simulations were run using the NAMD3^S8^. To obtain a suitable starting configuration, 100 ns and 20 ns equilibrations were carried out for the complex systems and the ligand systems, respectively.

For the production simulations, double-wide sampling (DWS) was used instead of the bidirectional simulation. In DWS, during a simulation with a specific λ, the free-energy changes corresponding to the forward and backward transformations are calculated simultaneously. 200 λ and 100 λ windows were set for the complex systems and the ligand systems, respectively. Each λ window comprised 0.4 ns of equilibration and 2 ns of data collection. The cumulative time required for the complex systems and the ligand systems amounted to 480ns and 240 ns, respectively. The BAR estimator in the BFEE2 package^S7^ was used for post-processing the absolute binding free energies.

Furthermore, Van der Waals (VDW) interactions are gradually decoupled over the λ range of 0.3–1.0 in alchemical transformations, whereas ligand decoupling entails linearly decreasing electrostatic interactions over the λ range of 0.0–0.5. For the formation of ligands, the opposite process is required. To prevent end-point disasters and enable the gradual switching of van der Waals contacts during alchemical transformations, a soft-core potential^S9-S11^ is used. The achievement of a hysteresis-based error smaller than or equal to *k*BT, or around 0.6 kcal/mol, is the convergence criteria for alchemical transformations.

At a time step of 4 fs, the simulations were carried out in the NPT ensemble. Langevin dynamics^S12^ with a friction coefficient of 5 ps^-1^ was employed to maintain the temperature at 300 K. The pressure at 1 atm was controlled using the Langevin piston Nosé-Hoover method^S13^. In order to truncate short-range van der Waals (VDW) and electrostatic interactions, a switching distance of 9 Å and a cutoff distance for bonding reactions of 11 Å were set up during equilibrium and production. Long-range electrostatic interactions were handled using the particle mesh Ewald (PME) method^S14–S16^, and water was restrained using the SHAKE algorithm^S4–S6^.

**References**

(S1) HMADA, C.; Belfon, K.; Ben-Shalom, I.; Berryman, J.; Brozell, S.; Cerutti, D.; Cheatham, T.; Cisneros, G.; Cruzeiro, V.; Darden, T., et al. Amber2022. San Francisco.: University of California 2022,

(S2) Maier, J. A.; Martinez, C.; Kasavajhala, K.; Wickstrom, L.; Hauser, K. E.; Simmerling, C. ff14SB: improving the accuracy of protein side chain and backbone parameters from ff99SB. *J. Chem. Theory Comput.* **2015**, *11*, 3696−3713.

(S3) GAFF and GAFF2 are public domain force fields and are part of the AmberTools16 distribution, available for download at http://amber.org (accessed October 2018).According to the AMBER development team, the improved version of GAFF, GAFF2, is an ongoing project aimed at “reproducing both the high quality interaction energies and key liquid properties such as density, heat of vaporization and hydration free energy”. GAFF2 is expected “to be an even more successful general purpose force field and that GAFF2-based scoring functions will significantly improve the successful rate of virtual screenings”.

(S4) van Gunsteren, W. F.; Berendsen, H. J. Algorithms for macromolecular dynamics and constraint dynamics. *Mol. Phys.* **1977**, *34*, 1311−1327.

(S5) Andersen, H. C. Rattle: A “velocity” version of the shake algorithm for molecular dynamics calculations. *J. Chem. Phys.* **1983**, *52*, 24−34.

(S6) Ryckaert, J.-P.; Ciccotti, G.; Berendsen, H. J. Numerical integration of the cartesian equations of motion of a system with constraints: molecular dynamics of n-alkanes. *J. Chem. Phys.* **1977**, *23*, 327−341.

(S7) Fu, H.; Chen, H.; Blazhynska, M.; Goulard Coderc de Lacam, E.; Szczepaniak, F.; Pavlova, A.; Shao, X.; C. Gumbart, J.; Dehez, F.; Roux, B.; Cai, W.; Chipot, C. Accurate determination of protein: ligand standard binding free energies from molecular dynamics simulations. *Nat. protoc.* **2022**, *17*, 1114−1141.

(S8) Phillips, J. C.; Braun, R.; Wang, W.; Gumbart, J.; Tajkhorshid, E.; Villa, E.; Chipot, C.; Skeel, R. D.; Kale, L.; Schulten, K. Scalable molecular dynamics with NAMD. *J. Comput. Chem.* **2005**, *26*, 1781−1802.

(S9) Zacharias, M.; Straatsma, T. P.; McCammon, J. A. Separation‐shifted scaling, a new scaling method for Lennard‐Jones interactions in thermodynamic integration. *J. Chem. Phys.*, **1994**, *100*, 9025−9031.

(S10) Beutler, T. C.; Mark, A. E.; van Schaik, R. C.; Gerber, P. R.; Van Gunsteren, W. F. Avoiding singularities and numerical instabilities in free energy calculations based on molecular simulations. *Chem. Phys. Lett.* **1994**, *222*, 529−539.

(S11) Pitera, J. W.; van Gunsteren, W. F. A comparison of non-bonded scaling approaches for free energy calculations. *Mol. Simul.* **2002**, *28*, 45−65.

(S12) Andersen, H. C. Molecular dynamics simulations at constant pressure and/or temperature. *J. Chem. Phys.* **1980**, *72*, 2384−2393.

(S13) Feller, S. E.; Zhang, Y.; Pastor, R. W.; Brooks, B. R. Constant pressure molecular dynamics simulation: The Langevin piston method. *J. Chem. Phys.* **1995**, *103*, 4613−4621.

(S14) Darden, T.; York, D.; Pedersen, L. Particle mesh Ewald: An N log (N) method for Ewald sums in large systems. *J. Chem. Phys.* **1993**, *98*, 10089−10092.

(S15) Essmann, U.; Perera, L.; Berkowitz, M. L.; Darden, T.; Lee, H.; Pedersen, L. G. A smooth particle mesh Ewald method. *J. Chem. Phys.* **1995**, *103*, 8577−8593.

(S16) Huang, Y.; Chen, W.; Wallace, J. A.; Shen, J. All-atom continuous constant pH molecular dynamics with particle mesh Ewald and titratable water. *J. Chem. Theory Comput.* **2016**, *12*, 5411−5421.
